# Supplementary figures and images for: High Levels of Anti-SARS-CoV-2 Receptor-Binding Domain (RBD) Antibodies One Year Post Booster Vaccinations among Hospital Workers in Indonesia: Was the Second Booster Needed?
Source: Vaccines (Basel). 2023 Jul 30;11(8):1300. doi: 10.3390/vaccines11081300 (PMC10457959; doi:10.3390/vaccines11081300)

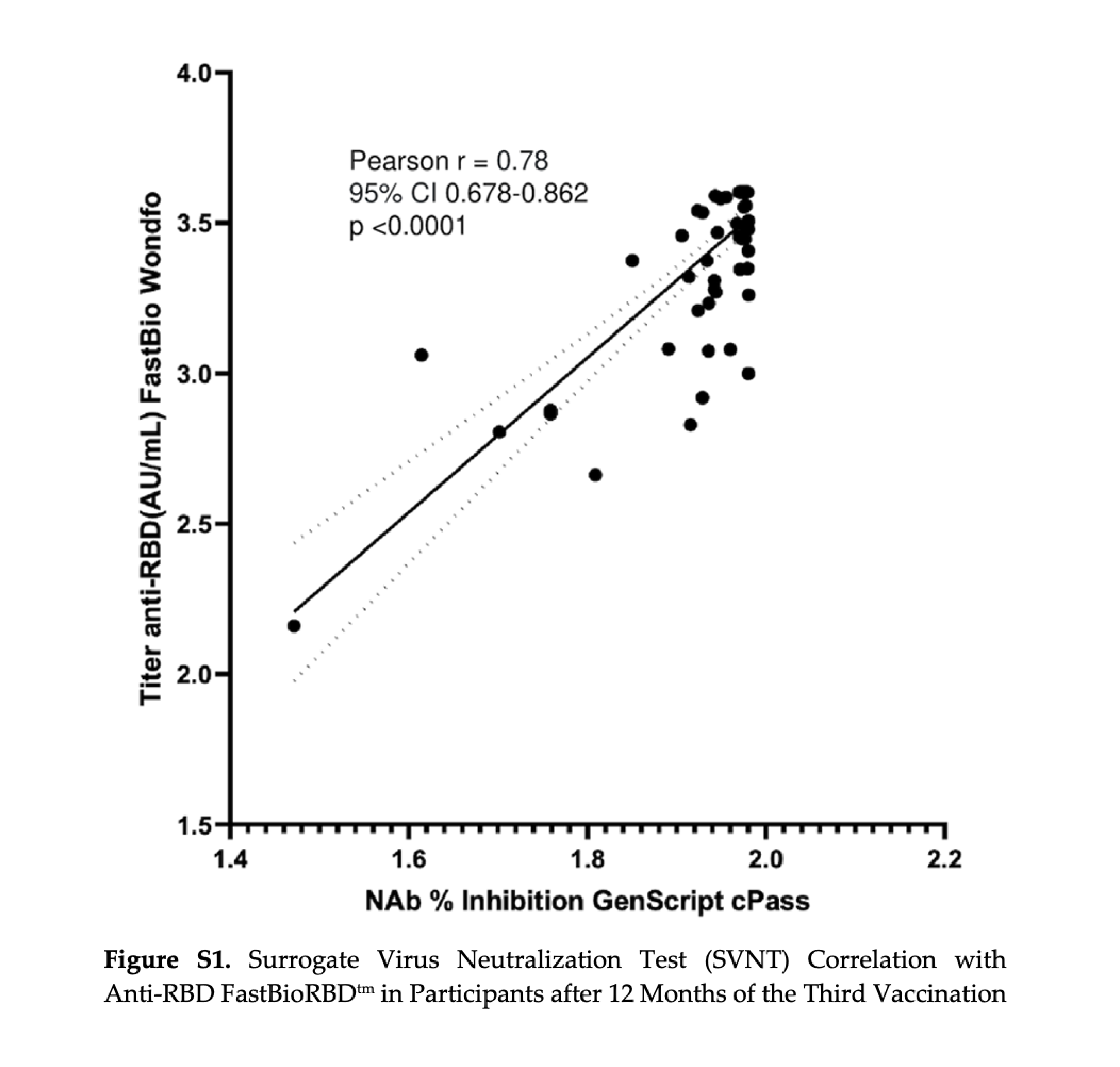

Supplement: Supplementary file 1 [file vaccines-11-01300-s001.zip › Supplementary Table and Figure/Supplementary Figure S1.tiff]
